# Potential Application of Gold Nanospheres as a Surface Plasmon Resonance Based Sensor for In-Situ Detection of Residual Fungicides

**DOI:** 10.3390/s20082229

**Published:** 2020-04-15

**Authors:** Hang Nguyen Thi Nhat, Ngoc Thuy Trang Le, Nguyen Thi Phuong Phong, Dai Hai Nguyen, Minh-Tri Nguyen-Le

**Affiliations:** 1 Faculty of Natural Sciences, Thu Dau Mot University, Thu Dau Mot City 820000, Binh Duong Province, Vietnam; hangntn@tdmu.edu.vn; 2 Institute of Research and Development, Duy Tan University, Danang 550000, Vietnam; lenthuytrang4@duytan.edu.vn; 3 Faculty of Chemistry, University of Science, Vietnam National University Ho Chi Minh City, 227 Nguyen Van Cu Street, District 5, Ho Chi Minh City 70000, Vietnam; ntpphong@hcmus.edu.vn; 4 Institute of Applied Materials Science, Vietnam Academy of Science and Technology, 01 TL29, District 12, Ho Chi Minh City 700000, Vietnam; nguyendaihai0511@gmail.com; 5 Graduate University of Science and Technology, Vietnam Academy of Science and Technology, 18 Hoang Quoc Viet, Cau Giay, Hanoi 100000, Vietnam; 6 Laboratory of Advanced Materials Chemistry, Advanced Institute of Materials Science, Ton Duc Thang University, Ho Chi Minh City 758307, Vietnam; 7 Faculty of Applied Sciences, Ton Duc Thang University, Ho Chi Minh City 758307, Vietnam

**Keywords:** gold, nanosphere, SERS, fungicides, Raman

## Abstract

It is essential to develop a simple and sensitive method to rapidly detect residual fungicides in agricultural products to protect human health. So far, little studies have been reported on potential application of gold nanospheres (AuNSps) as a surface plasmon resonance based sensor for in-situ detection of residual fungicides. Therefore, in this study, we investigated the potential application of AuNSps as a surface plasmon resonance based sensor for in-situ detection of fungicides. AuNSps were successfully synthesized via a seed-mediated method with some modifications. Firstly, gold nanoseeds were made during the reduction of chloroauric acid by trisodium citrate dihydrate (TSC). Then, AuNSps were grown from the seeds by using HAuCl_4_, TSC and EDTA. AuNSps were subsequently dropped on a glass substrate before covered by thiophanate methyl, a broad-spectrum systemic fungicide. The AuNSps coated glass substrate was subsequently dried in the air for further surface-enhanced Raman spectroscopy (SERS) measurements. Optical properties, shape and size of AuNSps were confirmed by UV-vis spectroscopy, XRD, SEM-EDX and TEM. The results showed that AuNSps were successfully synthesized with the size of 53 nm, and their resonance peak was located at 560 nm. The Raman signal intensity of thiophanate methyl covered on AuNSps is higher than that without AuNSps, indicating SERS effects of AuNSps deposited glass substrate.

## 1. Introduction

The development of science and technologies has led to the introduction of modern analytical methods with high sensitivity and accuracy, such as high performance liquid chromatography (HPLC), fluorescence assay (FL), high performance liquid chromatography mass spectrometry/mass spectrometry (HPLC–MS/MS), electrochemical assay (EC) and gas chromatography/mass spectrometry (GC/MS). However, these methods have some limitations such as complicated sample preparation and analysis processes that require operators to be highly qualified, time-consuming operation, high consumption of organic solvents and restricted on-site measurement. Since the discovery of Raman spectroscopy took placed in early 1928, Raman spectroscopy has been thought to be a promising method to overcome these disadvantages [1]. However, the application of Raman spectroscopy in analytical field was not popular at that time due to its low sensitivity as a result of low intensity of Raman scattered light as compared with the incident laser. In order to increase the intensity of Raman scattered light, a high-intensity laser should be used [2]. However, the sensitivity of the method is not really high until the study of surface-enhanced Raman spectroscopy (SERS) on the electrode silver anode of DL Jeanmaire was published in 1977 [3]. SERS is thought to be the result of the enhancement of Raman scattered light due to both physical and chemical factors [4]. The physical factor is the enhancement of the electromagnetic field on the nanoparticle surface due to the surface plasmon resonance, while the chemical factor is the electrical resonance and charge transfer between the adsorbent molecules and the metal surface [5]. SERS can be observed on the surface of several of nanoparticles such as Au, Ag, Pt, Cu and Pd [6,7,8,9,10,11]. Among them, gold nanoparticles with non-toxicity and high bioactivity have gained much interest, and they present a wide range of applications in modern medical and biological studies such as drug, DNA and antigens delivery; biosensorics, genomics, immunoanalysis, detection of microorganisms and cancer cells and bioimaging [12]. It has been argued that the shape, size and structure of gold nanoparticles determine their chemical and physical properties [13,14]. Therefore, shape-, size- and structure-controlled synthesis of gold nanoparticles is urgently important and considerably challenging. To date, possible wet synthesis methods have been used to fabricate gold nanoparticles of various controlled sizes, shapes and structures such as nanospheres, nanorods, nanoshells, nanocages, nanostars, etc. [15].

**Figure 1:**
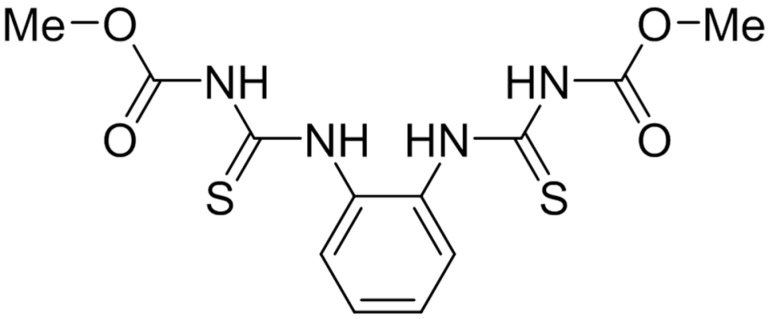
The chemical structure of thiophanate methyl.

Thiophanate methyl whose chemical structure is shown in Figure 1 is known as a member of the benzimidazole group of fungicides [16]. It is a broad-spectrum systemic fungicide that has been employed to control the pathogens recently. However according to the office of Environmental Health Hazard Assessment (OEHHA), long exposure to residue from thiophanate methyl in agricultural products may cause serious effects on human health [16]. Therefore, it is essential to develop a sensitive on-site method to detect residual thiophanate methyl in agricultural products to prevent humans from being exposed to thiophanate methyl [17]. The analysis of thiophanate methyl residues by Au nanospheres (AuNSps)—supported Raman spectroscopy—has the potential to be an accurate portable analytical method. So far, little studies have been reported on the potential application of AuNSps as a surface plasmon resonance based sensor for in-situ detection of residual fungicides.

In this work, AuNSps were synthesized by a facile method using TSC as a reducing agent and EDTA as a protective agent during the nucleation stage. The as-synthesized AuNSps were used as a platform to investigate the SERS signal in thiophanate methyl fungicide analysis.

## 2. Materials and Methods

### 2.1. Chemicals and Characterization

Tetrachloroauric (III) acid trihydrate (HAuCl_4_·3H_2_O, ≥99.5%) and ethylenediaminetetraacetic acid (EDTA, ≥98.0%) were purchased from Merck (Darmstadt, Germany). Trisodium citrate dihydrate (TSC, ≥99.0%) were purchased from Prolabo (Kennersburg, NJ, USA). Deionized (DI) water (>18 MW, Millipore, Darmstadt, Germany) was used during the synthesis of AuNSps. A commercial product, called TOPSIN M 70WP (containing 70% of thiophanate methyl), was purchased from Nippon Soda Company, Ltd. All reagents were used without further purification.

The morphology of the as-synthesized samples was obtained by scanning electron microscopy (Hitachi, Horiba S-4300) coupling with energy-dispersive X-ray (EDX) spectroscopy operated at 20 kV of the incident electron beam energy. Transmission electron microscope (TEM) images were obtained with a JEOL JEM-1400 (120 kV). X-ray diffractometer (XRD, Bruker D8 advance powder diffractometer model) with Cu K*α* radiation (*λ* = 1.54056 Å) operated at 40 kV and 30 mA with a scanning rate of 0.02° per step in the 2*θ* range of 10° ≤ 2*θ* ≤ 90° was carried out to characterize the crystal structure of AuNSps. UV-vis spectroscopy (UV-Vis-NIR-V670, JASCO, Tokyo, Japan) was utilized to investigate the optical properties of AuNSps. The Raman spectra of AuNSps samples were recorded on a LabRam HR micro-Raman instrument with a 532 nm Ar+ ion laser at room temperature.

### 2.2. Synthesis of Au Nanoseeds and AuNSps

Au nanoseeds were synthesized by the Turkevich method [15] with some modifications. Firstly, 100 µL of 25 mM HAuCl_4_ was mixed with 10 mL of DI water, and the obtained solution was boiled at 100 °C. Under vigorous stirring 300 µL of 1% TSC was then added to the solution. The color of the solution will change from colorless to reddish. After cooling at room temperature, the solution was kept in dark for 30 min for seeding crystallization of Au nanoseeds.

**Figure 2:**
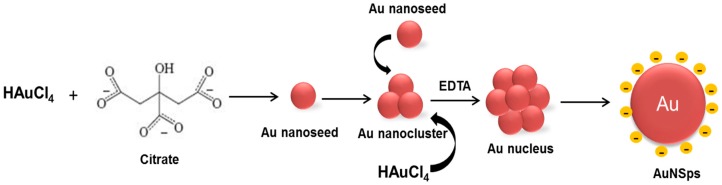
Fabrication process of gold nanospheres (AuNSps).

For the synthesis of AuNSps, 50 µL of the Au nanoseed solution and 100 µL of 25 mM HAuCl_4_ were firstly added into 100 mL of DI water under vigorous stirring. Next, 20 µL of 1% TSC and 100 µL of 0.1 M EDTA were added to the mixture. The solution was kept in the dark for 30 min for stabilization. The whole procedure of AuNSps synthesis was illustrated in Figure 2.

### 2.3. SERS Measurement

**Scheme 1:**
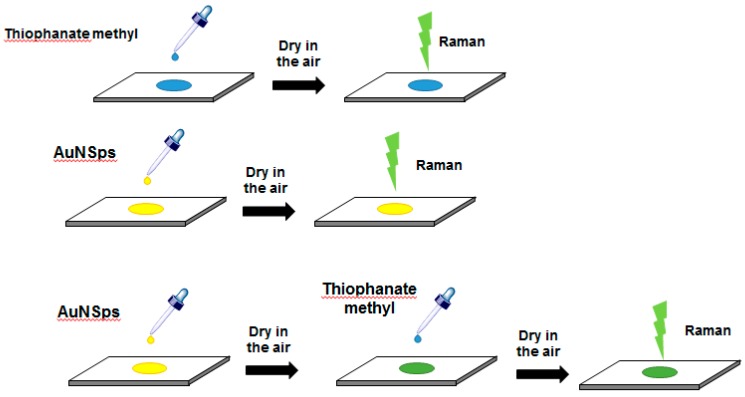
Surface-enhanced Raman spectroscopy (SERS) measurement procedure.

The Raman spectra of thiophanate methyl were recorded on an AuNSps coated glass substrate in order to study the SERS effects. To prepare AuNSps coated glass substrate, the substrate was firstly rinsed with a 3:1 (v/v) mixture of concentrated H_2_SO_4_ and 30% H_2_O_2_ in 15 min, subsequently washed with DI water and dried in the air. Twenty microliters of AuNSps solution was gently dropped onto the clean substrate homogeneously, and the substrate was then dried in the air. Next, Twenty microliters of thiophanate methyl (in DI water) was gently dropped onto the AuNSps coated glass substrate, and the substrate was subsequently dried in the air for further SERS measurements. For comparison, Raman spectra of a clean substrate, AuNSps and thiophanate methyl solutions (10^−4^ M and 0.1 M) were also recorded. The procedure of SERS measurement was illustrated in Scheme 1.

## 3. Results and Discussion

### 3.1. Synthesis of AuNSps

**Figure 3:**
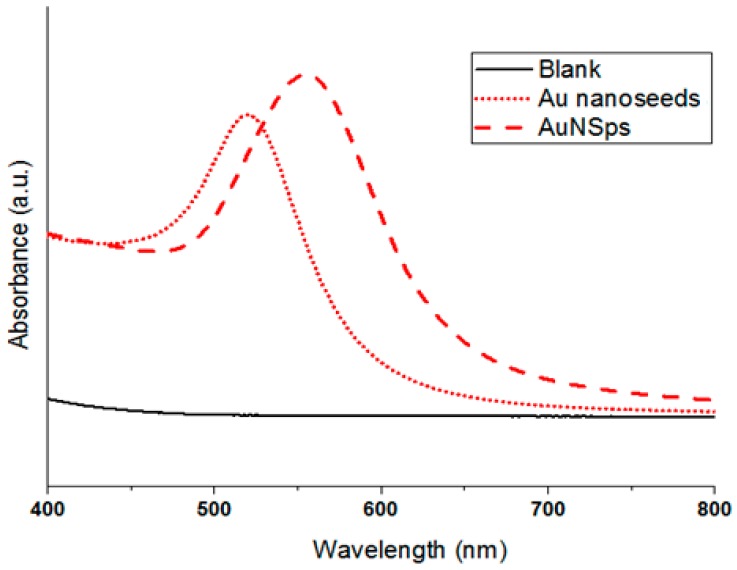
UV-Vis spectra of Au nanoseeds and AuNSps.

Optical properties of AuNSps and Au nanoseeds were studied through UV-Vis spectra. As shown in Figure 3, the spectra of Au nanoseeds exhibited a characteristic peak at 521 nm, while the resonance peak of AuNSps located at 560 nm [18]. The red-shift of surface plasmon resonance peak indicated the crystal growth of Au nanoparticles from Au nanoseeds. It is widely accepted that the optical properties of AuNSps are highly dependent on the nanoparticle diameter [19]. Smaller nanospheres primarily absorb light and have absorption peaks near 520 nm, while larger spheres exhibit light scattering and have significantly broaden and red-shifted absorption peaks. Larger spheres scatter more light both because they have larger optical cross sections, and also because the ratio of scattering to total extinction increases with size [20,21].

**Figure 4:**
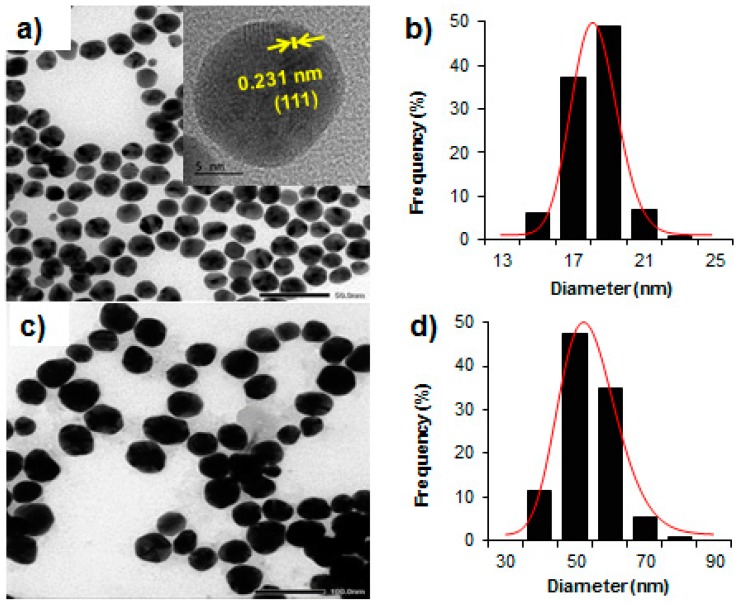
TEM image and particle size distribution of (**a**,**b**) Au nanoseeds, and (**c**,**d**) AuNSps. The inset: HRTEM image of Au nanoseeds.

The solution of Au nanoseeds consisted of clusters of Au nanosized particles with the average diameter of 18.27 ± 0.08 nm (Figure 4a,b). Nanoseeds with the crystalline lattices of 0.231 nm, corresponding to the (111) crystal plane of Au, is clearly observed in the HRTEM image (Figure 4a, the inset) [22,23]. The TEM images of AuNSps revealed monodisperse spherical nanostructures with the average diameter of 53.52 ± 0.36 nm (Figure 4c,d).

**Figure 5:**
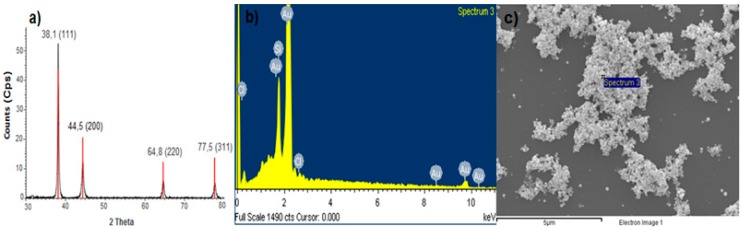
(**a**) XRD spectra of AuNSps, (**b**) EDX spectra and (**c**) SEM image of AuNSps coated glass.

Figure 5a revealed XRD pattern of AuNSps with characteristic peaks of Au at 2*θ* = 38.1° (111), 44.3° (200), 64.5° (220) and 77.7° (311) [24]. No impurities were found in XRD spectra of AuNSp. The EDX spectrum in Figure 5b represented that Au and Si were the major elements of AuNSps coated glass substrate, indicating successful coating of the glass substrate with AuNSps.

### 3.2. SERS Measurement

**Figure 6:**
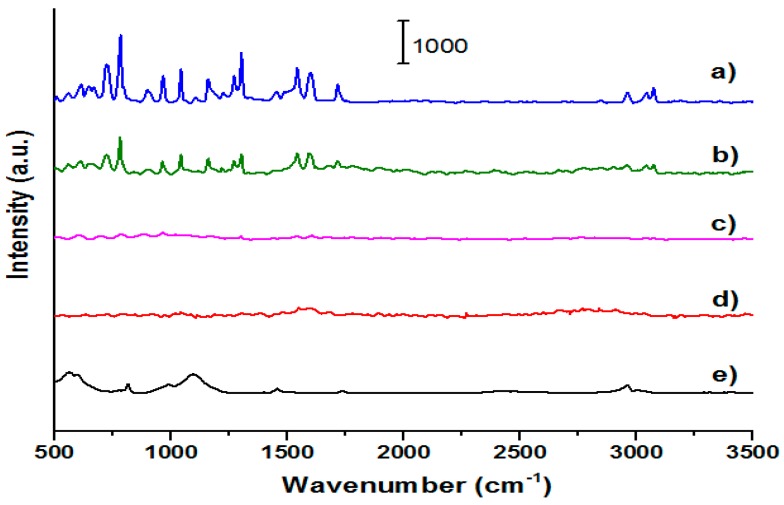
Raman spectra of (**a**) 0.1 M thiophanate methyl, (**b**) 10^−4^ M thiophanate methyl deposited onto AuNSps coated glass substrate, (**c**) 10^−4^ M thiophanate methyl, (**d**) AuNSps, and (**e**) glass substrate.

The Raman spectra of 0.1 M thiophanate methyl in Figure 6a showed the signals at 613 cm^−1^, 726 cm^−1^, 779 cm^−1^, 898 cm^−1^, 959 cm^−1^ and 1538 cm^−1^, which are ascribed to -N-C=S deformation, N-H wagging of –(C=O)-NH-(C=S)-, C=S stretching, C-S stretching, C=S and C-N, respectively. Moreover, other signals of =C-H deformation, N-C-N asymmetric stretching, C-O, C-O-C, C=C, C=O and N-H were also observed at 1039 cm^−1^, 1154 cm^−1^, 1267 cm^−1^, 1298 cm^−1^, 1601 cm^−1^ and 1708 cm^−1^, respectively [25]. As shown in Figure 6d,e, the glass substrate or AuNSps itself did not give any Raman signals of thiophanate methyl indicating thiophanate methyl-free surfaces. It should be noted that even though in the presence of thiophanate methyl, those signals could not be observed when the concentration of thiophanate methyl was as low as 10^−4^ M (Figure 6c), probably due to being under the detection limit. Interestingly, at the same concentration of thiophanate methyl, most of the characteristic Raman peaks of thiophanate methyl were clearly observed after the deposition of thiophanate methyl onto the AuNSps coated glass substrate (Figure 6b), indicating the SERS effect of AuNSps. Therefore, this effect is useful for the detection at a low level of thiophanate methyl, particularly under the detection limit of thiophanate methyl.

**Figure 7:**
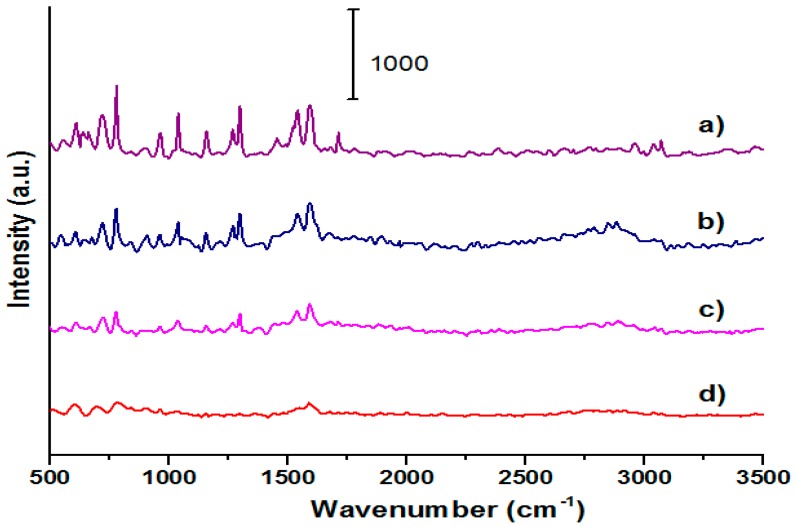
Raman spectra of thiophanate methyl collected on AuNSps coated glass substrate at different concentrations of thiophanate methyl: (**a**) 30 µM, (**b**) 23 µM, (**c**) 17 µM and (**d**) 11 µM.

To elucidate the sensitivity of the method, 20 µL of thiophanate methyl solutions with concentrations ranging from 11 to 30 µM were deposited onto AuNSps coated glass substrates, which serve as Raman probes. The corresponding Raman spectra of thiophanate methyl were collected in Figure 7. It can be seen that the characteristic peaks of thiophanate methyl could be clearly identified in all Raman spectra. Typically, the intensity of SERS signals increased with the increasing investigated concentrations of thiophanate methyl. The limit of detection (LOD) was determined as low as 17 µM at which the signal to noise (S/N) ratio for the peak 779 cm^−1^ dropped to 3. The limit of quantification (LOQ), therefore, was estimated as 57 µM.

**Figure 8:**
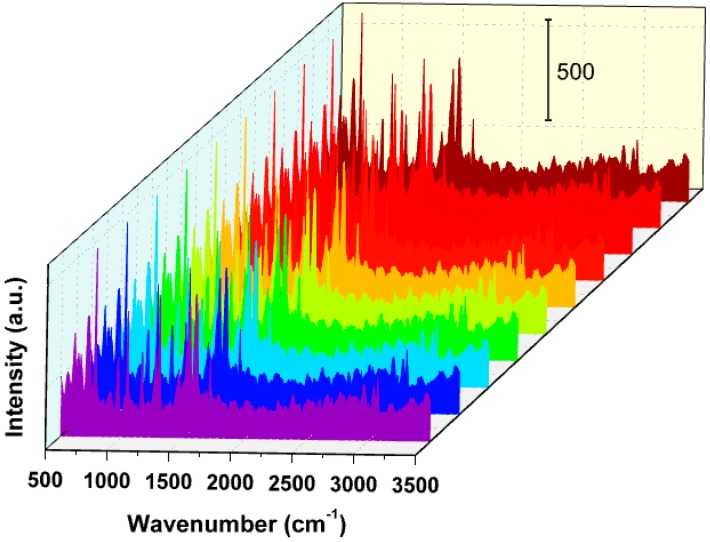
Raman spectra of 30 µM thiophanate methyl collected at 10 random locations on AuNSps coated glass substrate.

The reproducibility of the measurement was also evaluated by collecting SERS spectra of 30 µM thiophanate methyl at 10 random locations on the as-fabricated substrate, as shown in Figure 8. The relative standard deviation (RSD) was found to be 4.8%, which indicates a good reproducibility for routine SERS analysis.

## 4. Conclusions

AuNSps were successfully synthesized from crystallization of Au nanoseeds through a facile seed-mediated method using TSC as a primary reducing agent and EDTA as a protective agent during the nucleation stage. The as-synthesized AuNSps showed SERS effect for thiophanate methyl with low LOD of thiophanate methyl down to 17 µM. The Raman signal intensity of thiophanate methyl covered on AuNSps was higher than that without AuNSps, which revealed the potential application of AuNSps as a surface plasmon resonance based sensor for in-situ detection of fungicides with high sensitivity and reproducibility.
